# Paraganglioma of the Filum Terminale: Case Report, Pathology and Review of the Literature

**DOI:** 10.7759/cureus.354

**Published:** 2015-10-15

**Authors:** Maziyar A Kalani, Steven D. Chang, Brandon Vu

**Affiliations:** 1 Department of Neurosurgery, Stanford University School of Medicine

**Keywords:** spinal paraganglioma, spine tumor, intradural tumor, spine surgery

## Abstract

Spinal paragangliomas are very rare neuroendocrine tumors often presenting with low back pain and radicular symptoms; once resected, they often show benign clinical outcomes. Radiographically spinal paragangliomas mimic more commonly described tumors, such as ependymomas, schwannomas, meningiomas, and even hemangiomas, but a “salt and pepper” appearance related to a serpiginous vascular structure is instructive. Indeed, the rarity of this tumor makes the diagnosis rather challenging radiographically. Graded as a WHO Grade I tumor, they are slow-growing with low proliferation indices. Gross total resection is the mainstay of operative treatment but is often limited by tumor adherence to functional nerves. Here, we present a case of this rare tumor and its management, including a review of the pathology and literature related to this tumor.

## Introduction

Paragangliomas are neoplasms originating from the autonomic nervous system, found generally in adrenal and extra-adrenal locations [1]. Extra-adrenal paragangliomas are rare and occur most commonly in the carotid bodies and the jugular glomus [2-3]. Primary spinal paragangliomas are extremely rare, most frequently involving the cauda equina and the filum terminale [1-2, 5-6, 8, 11-18, 22, 24-25, 28]. Legacé, et al. first described them in 1978 in a case report describing a filum terminale lesion with histologic characteristics of proliferating lobules and sheets of regular cells within a rich vascular network. It was speculated that the glomus coccygeum was the origin of this tumor [19].

## Case presentation

This is a case of a 54-year-old man with a history of diabetes mellitus Type 2 and hypertension with a four-month history of back pain, radiating to his buttocks, worse on the right compared to the left. He denied any weakness, numbness, or tingling in his lower extremities. The pain was rated 10/10 upon initial presentation but decreased to 3/10 upon presentation for neurosurgical evaluation. Two months after the initial onset of pain, he was bedbound and incapacitated, unable to fulfill activities of daily living.

Other than back pain, this patient complained of urinary hesitancy upon initiation and bearing down to initiate bowel movements caused a lancinating pain in his back. He denied any changes in perineal sensation but has had erectile dysfunction, which has been attributed to low testosterone for which is he undergoing replacement therapy.

This patient presented to our clinic in May 2015 with a complaint of minimal back pain and a localized patch of numbness on the lateral aspect of his right leg. His neurologic review was negative. His social habits were negative for illicit drug use or alcohol use; he is a former smoker of 22.5 pack-years and denies any exposures. Family history was negative for schwannomas, neurofibromas, or other brain and spinal cord tumors; his father died of renal cell carcinoma. He reports having had multiple skin and subcutaneous lesions in his arms, legs, and trunk, one of which was biopsied and diagnosed as benign without further specification.

On physical examination, he demonstrated normal vital signs. His heart, lung, abdominal, and skin examinations were normal. Cranial nerves, 2-12, were intact as was his motor exam in all four extremities. He experienced a patch of numbness on the lateral aspect of his right calf. His reflexes were 2+ throughout and without clonus. His gait was narrow-based and normal.

**Figure 1:**
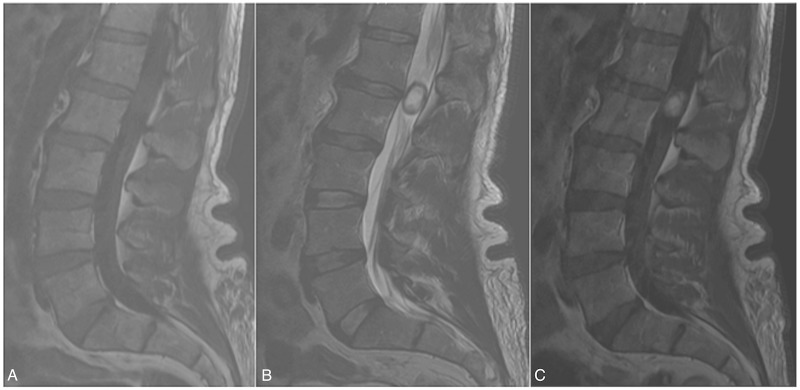
Magnetic Resonance Imaging (MRI) of the Lumbar Spine Magnetic Resonance Imaging without and without contrast a. T1-weighted imaging (T1WI) demonstrating a hypo-/isointense lesion at the L1-2 disc space which is b. hyperintense but with c. faint, homogeneous enhancement on T1WI with contrast

Neuroimaging with an MRI of the lumbar spine with and without contrast demonstrated an intradural mass at the level of the conus measuring 1.4 x 1.1 x 2.2 cm with heterogeneous contrast enhancement (Figure 1). Small disc herniations were also noted at L2-3 and L4-5.

The clinical and radiographic evaluation was concerning for a neurogenic tumor at the level of the conus, likely a neurofibroma, schwannoma, or possibly an ependymoma. Given his history of multiple skin and subcutaneous lesions, the possibility of a unifying diagnosis of neurofibromatosis was entertained and recommended surgical resection for the purposes of decompression of his nerve roots as well as to obtain pathology. After signing informed consent, he underwent resection.

### Operative plan

Once the decision for operative resection was made, the patient was taken to the operating room for an L1-2 laminectomy for resection of this L1-2 intradural extramedullary spinal tumor.

The patient underwent standard anesthetic induction and intubation, followed by prone positioning. The intended L1-2 space was localized using fluoroscopy and a subperiosteal dissection was performed, followed by an L1-2 laminectomy. A midline dural opening exposed the tumor which was readily visible using the operative microscope. A nerve stimulator was used to identify the nerve roots. The tumor was then debulked using the CUSA ultrasonic aspirator (Integra, Plainsboro, NJ), and the microscissors. 

Upon stimulating the tumor capsule, it was apparent that the S1 nerve roots were adherent to the tumor, and thus, the decision was made to leave some residual tumor in order to preserve S1 nerve function. Therefore, a near gross total resection was completed (1-2% residual along the S1 roots), and a dural closure was performed after meticulous intradural hemostasis was obtained. Hemostasis was then achieved in the surgical cavity and copious antibiotic irrigation was applied. The muscle, fascia, subdermal, and dermal layers were subsequently closed in a standard fashion.

The patient was then turned to the supine position and extubated. His immediate postoperative examination demonstrated a stable preoperative exam, including 5/5 strength in his bilateral lower extremities. 

### Pathology

**Figure 2:**
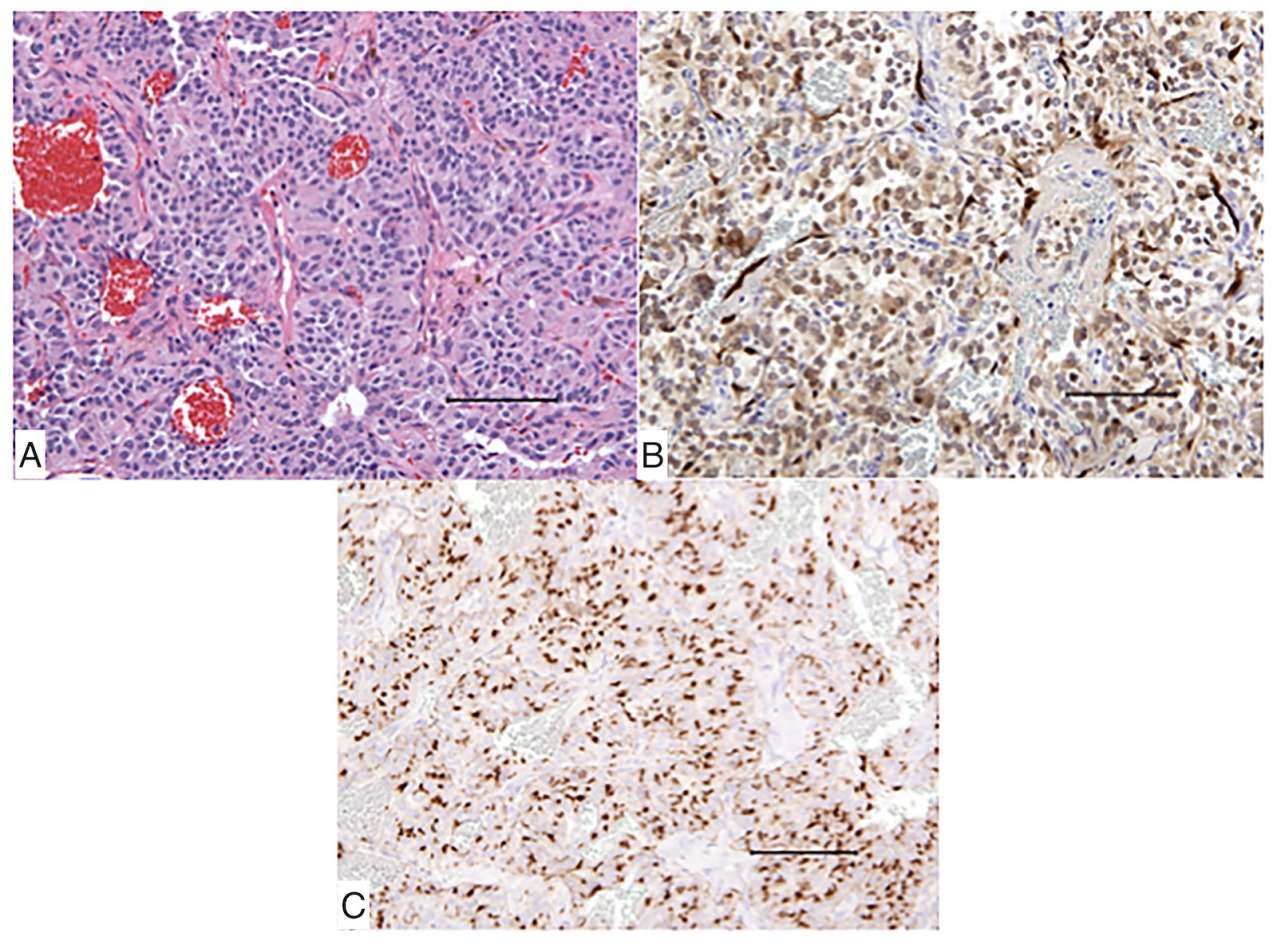
Histopathologic Findings a. Microscopic analysis at 200x magnification using hematoxylin and eosin (H&E) stain as well as b. S100 stain and c. Chromogranin stain, all at 200x magnification

Histologic sections demonstrated a neuroendocrine neoplasm with architectural features of a paraganglia, including Zellballen configurations of neoplastic neuroendocrine cells surrounded by delicate vessels and occasional sustentacular cells (Figure 2).

The neoplastic cells showed amphophilic to mildly eosinophilic cytoplasm and regular round nuclei with finely stippled chromatin. A few scattered mitotic figures were seen. Immunostains for synaptophysin, chromogranin, and S100 were positive in the neoplastic cells. S100 highlights scattered few sustentacular cells and nerve fibers with intense staining.

### Postoperative evaluation

Patient MA was seen three weeks postoperatively in our neurosurgery outpatient clinic with minimal incisional pain and muscle spasms. He had self-titrated on narcotics and antispasmodics with a desire to return to work. Since resection, his initial preoperative complaint of right leg pain and numbness had resolved. He denied any paresis, numbness, or incontinence.

On neurological assessment, his examination was intact. Postoperative imaging was deferred at the time of this postoperative evaluation with a plan to undergo magnetic resonance imaging at the three-month postoperative time point; at that time, should progression be seen, we would consider stereotactic radiosurgical boost to the small residual at the S1 nerve. He was advised that he could return to light work and activity and begin physical therapy.

## Discussion

Paragangliomas are neoplasms originating from the autonomic nervous system, found generally in adrenal and extra-adrenal locations [1]. Extra-adrenal paragangliomas are rare and occur most commonly in the carotid bodies and the jugular glomus [2-3]. Primary spinal paragangliomas are extremely rare, most frequently involving the cauda equina and the filum terminale [1-2, 4-17]. Legacé, et al. first described them in 1978 in a case report describing a filum terminale lesion with the histologic characteristic of proliferating lobules and sheets of regular cells within a rich vascular network. It was speculated that the glomus coccygeum was the origin of this tumor [18].

Paragangliomas are classified as World Health Organization (WHO) Grade I given their characteristically slow growth and histologically benign appearance. As they are neurogenic and arising from the autonomic nervous system, they are sub-classified into sympathetic – secreting catecholamines – or parasympathetic and thus functionally non-secretory [4, 19]. In the central nervous system, 80-90% of paragangliomas are identified in at the bifurcation of the common carotid artery and in the middle ear and predominantly of the parasympathetic type. These are predominantly parasympathetic without remarkable symptoms [20].

As a subset, spinal paragangliomas are even less common and generally occur at the lumbosacral extent of the spine without reported variation in distribution [2, 4]. There is no specific decade of highest prevalence but a male to female ratio of nearly 1.7:1 has been reported [1, 17]. The average duration of symptoms preceding diagnosis is typically around two years on the short end of the interval [17, 21]. Low back pain and sciatica were the most common clinical presentations in the present study, and sensory-motor and sphincter dysfunction can develop along with clinical progression [1, 15, 17]. Some cases can present with symptoms of radiculopathy or slowly progressive spinal cord compression [16]. Individual case reports have shown associations with superficial siderosis [6, 22] and even elevated intracranial pressure [12, 23].

Preoperative diagnosis is determined using magnetic resonance imaging, but due to the rarity of paragangliomas, radiographic diagnosis is challenging. Early on, the MRI characteristic of paragangliomas had been described [24-29]. On MRI, paragangliomas are generally hypointense to isointense on T1WI and isointense to hyperintense on T2WI; T1WI homogeneity in contrast enhancement helps to delineate the tumor [17, 30]. A characteristic “salt & pepper” appearance on T2WI is often described resulting from a rich vascular nature of paragangliomas; serpiginous flow voids are often evident on T2WI and occasionally peripheral hemosiderin is evident as a hypointensity in the T2WI. Radiographic differential diagnoses include myxopapillary ependymoma, schwannoma, meningioma, teratoma, and hemangioma [1].

Surgical pathology provides the definitive diagnosis of resected lesions. A “Zellballen” pattern as a solid mass of the paraganglion-type nests or cords is often described [2-3, 15]. Immunohistochemical staining is often S-100 positive while Ki-67 proliferative markers are generally low, suggesting a benign tumor of neuronal origin [4, 21].

Long-term follow-up data for management of spinal paragangliomas is sparse. In the largest analysis of spinal paragangliomas, Xu and colleagues followed 19 cases and determined the mean age of presentation to be 47.7 years. Initial presenting symptoms typically included back pain with sciatica, which immediately resolved with operative resection; three of five cases with incomplete resection showed recurrence of disease at 62.1 months [17]. Gross total resection is advocated but sometimes found difficult due to adhesion to functional nerve roots [17].

Data for adjunct treatment modalities also remains sparse. Conventional radiotherapy affords control rates of 90-100% for intracranial paragangliomas and is often advocated as a first-line therapy for asymptomatic patients with intracranial paragangliomas [31-33].

## Conclusions

Spinal paragangliomas are a very rare entity, often presenting with low back pain and radiculopathic symptoms. Their clinical course is generally benign with operative resection as the first-line treatment with nearly immediate improvement in symptoms. Radiographic characteristics include a vascular, “salt and pepper” appearance with prodigious flow voids and an occasional hemosiderin rim. Gross total resection is favored when possible with the realization that often-functional nerve roots may complicate the ability to fully resect the lesion. The role of adjunctive radiotherapy is unknown but, borrowing from the intracranial paraganglioma literature, may be useful in long-term progression management.
